# Tunable Graphene Phononic Crystal

**DOI:** 10.1021/acs.nanolett.0c04986

**Published:** 2021-02-23

**Authors:** Jan N. Kirchhof, Kristina Weinel, Sebastian Heeg, Victor Deinhart, Sviatoslav Kovalchuk, Katja Höflich, Kirill I. Bolotin

**Affiliations:** † Department of Physics, Freie Universität Berlin, Arnimallee 14, 14195 Berlin, Germany; ‡ Ferdinand-Braun-Institut gGmbH Leibniz-Institut für Höchstfrequenztechnik, Gustav-Kirchhoff-Strasse 4, 12489 Berlin, Germany; § Helmholtz-Zentrum Berlin für Materialien und Energie, Hahn-Meitner-Platz 1,14109 Berlin, Germany

**Keywords:** Nanomechanics, phononic crystal, graphene, optomechanics, resonators, NEMS

## Abstract

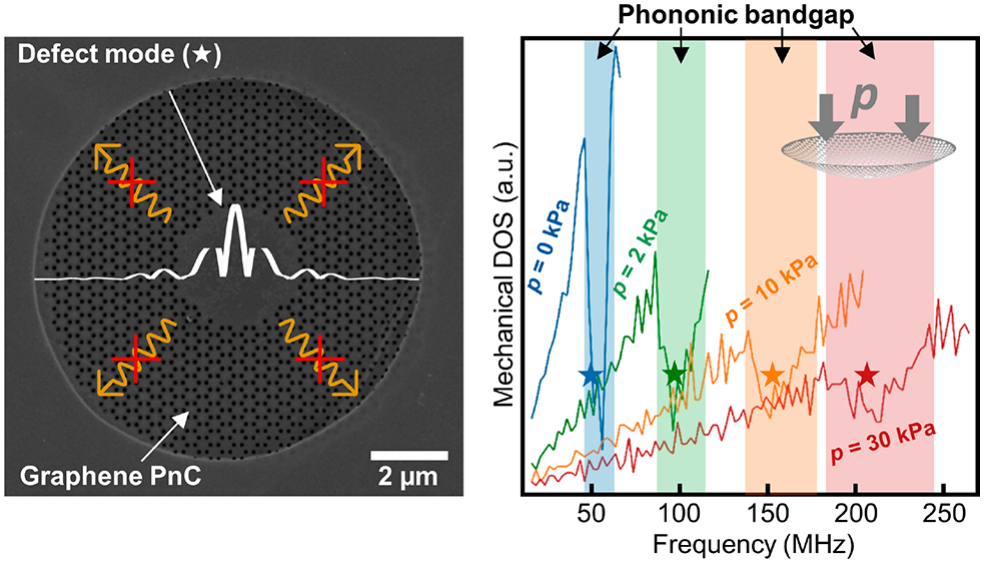

In the field of phononics, periodic patterning controls vibrations and thereby the flow of heat and sound in matter. Bandgaps arising in such phononic crystals (PnCs) realize low-dissipation vibrational modes and enable applications toward mechanical qubits, efficient waveguides, and state-of-the-art sensing. Here, we combine phononics and two-dimensional materials and explore tuning of PnCs via applied mechanical pressure. To this end, we fabricate the thinnest possible PnC from monolayer graphene and simulate its vibrational properties. We find a bandgap in the megahertz regime within which we localize a defect mode with a small effective mass of 0.72 ag = 0.002 m_physical_. We exploit graphene’s flexibility and simulate mechanical tuning of a finite size PnC. Under electrostatic pressure up to 30 kPa, we observe an upshift in frequency of the entire phononic system by ∼350%. At the same time, the defect mode stays within the bandgap and remains localized, suggesting a high-quality, dynamically tunable mechanical system.

## Introduction

A phononic crystal (PnC) is an artificially manufactured structure with a periodic variation of material properties, for example, stiffness, mass, or stress.^1^ This periodic perturbation creates a meta-crystallographic order in the system leading to a vibrational band structure hosting acoustic Bloch waves in analogy to the electronic band structure in solids.^1^ Designing the lattice parameters of the meta-structure allows one to directly manipulate phonons at various length scales.^2−4^ This can be used to guide^5−7^ and focus phonons^8,9^ or to open a vibrational bandgap.^1,10−12^

Phononic bandgaps in periodic structures suppress radiation losses and allow for highly localized modes (of frequency *f*) on artificial irregularities.^13,14^ The quality factors () of these so-called defect modes are especially high.^15,16^ In particular, resonances with *Q* > 8 × 10^8^ have been observed at room temperature in silicon nitride (SiN) PnCs.^15−17^ In these devices, the quality factor exceeds the empirical *Q* ∼ *m*^1/3^ rule,^17−19^ and the vibrational periods overcome the thermal decoherence time limit of τ = *hQ*/*k*_B_*T*.^15,17^ This, in turn, enables the study of quantum effects in resonators of macroscopic size, all at room temperature.^20,21^

Frequency tunability in PnCs could add an unprecedented knob to control a broad range of phononic application and thereby provides access to new regimes of guiding, filtering, and focusing phonons.^22−33^ It would furthermore allow one to resonantly couple to an external optical or mechanical excitation and thus realize sensing applications with mechanical qubits and studies on quantum entanglement.^34^ Yet, the mechanical resonances in PnCs are determined by material constants and the crystal geometry.^22,23,26−28^ In principle, the mode frequencies can be controlled by changing the temperature^29,30^ or by an external magnetic field.^31,32^ This, however, only provides limited tunability and necessitates heating the system or inclusion of magnetic materials. While SiN, as well as other conventional low-loss materials, is very stiff and allows only limited mechanical tunability,^24,33^ strain has been used to adjust the frequency response of elastic polydimethylsiloxane (PDMS).^25^ Unfortunately, low crystalline quality of that material led to limited tunability and very small *Q*s for mechanical modes.

Recently, PnCs made from two-dimensional (2D) materials have been considered.^35−37^ Such materials feature intrinsically low mass, high fundamental frequency, and easily accessible displacement nonlinearity. Most importantly, their high tensile strength and monolayer character allows the ability to mechanically strain them up to 10%.^38^ That invites consideration of mechanically controllable 2D-material based PnCs. Specifically, we expect the entire acoustic band structure of such a PnC to be highly tunable by applying mechanical pressure. Nevertheless, tunability of 2D phononic systems as well as localized defect modes in them have not been studied yet.

Here, we investigate mechanical tunability in a realistic graphene PnC. We fabricate a suspended micron-sized monolayer graphene PnC via focused helium ion beam milling (FIB) and characterize it spectroscopically. We then use experimentally established parameters to calculate the phononic band structure of the resulting PnC. We find a phononic bandgap from 48.8 to 56.5 MHz inside of which we localize a defect mode with an effective mass of 0.72 ag. Finally, we computationally investigate the mechanical tunability of the PnC under pressure induced by a local electrostatic gate.^39,40^ The applied pressure smears out the phononic bandgap as the out-of-plane displacement breaks the symmetry and causes perturbations of the artificial lattice, yet the mode shape of the defect mode remains highly localized. Overall, we can tune the resonance frequency of the defect mode by more than 350% and access new regimes of strain engineering.

## Results

### Designing a Tunable Phononic Crystal

**Figure 1:**
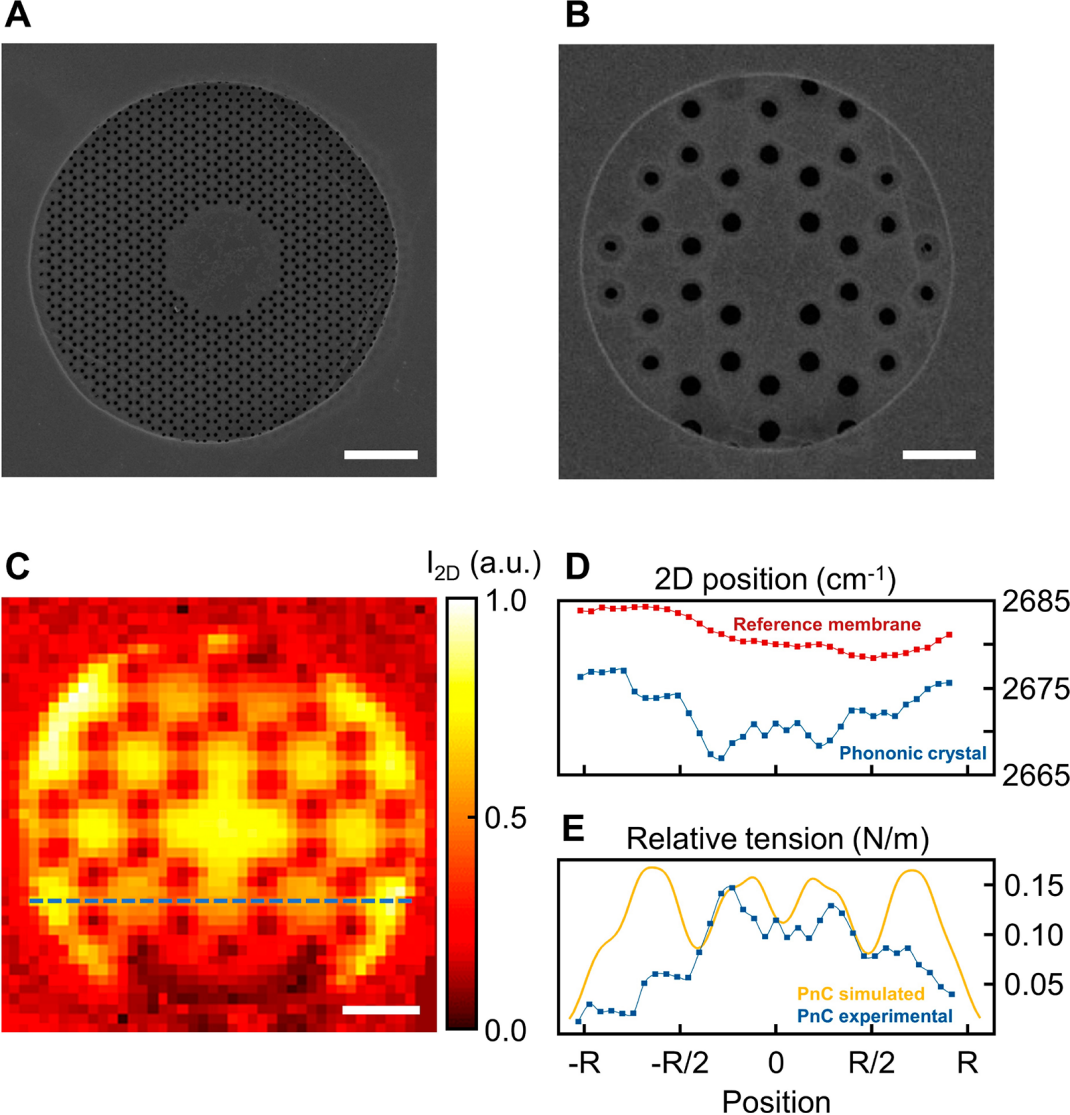
Graphene phononic crystals and tension redistribution. (A,B) Helium ion micrographs of prototype monolayer graphene phononic crystal devices with lattice constants 350 nm and 2 μm, respectively. Scale bar length is 2 μm. The phononic pattern, a honeycomb lattice of holes with a defect in its center, allows us to localize a vibrational defect mode. The ringlike features around the holes in (B) are due to incomplete removal of graphene most likely caused by contamination (details in Supporting Information). (C) Intensity map of the Raman-active 2D mode of graphene for the device shown in (B). The periodic pattern is clearly visible. (D) Raman 2D-mode position along a line cut (dashed line in (C)) for a PnC (blue) and reference membrane (red). The PnC shows a periodic variations of much larger amplitude compared to the fluctuation in the reference sample. (E) Comparison of the relative tension extracted from Raman measurements (blue) to the simulated tension distribution (yellow) confirming the redistribution of tension upon pattering. The simulation includes spatial broadening due to the finite size of the laser spot.

Our device design of a tunable, two-dimensional PnC consists of the following key elements. First, the PnC material must be freestanding to allow out-of-plane displacement. Second, it is necessary to use an electrically conductive material. In that case, an electrostatic gate electrode can be used to apply pressure and to induce tension as the membrane is pulled toward the gate. Third, the material needs to be flexible to allow large mechanical tunability with small pressures. Monolayer graphene with its high carrier mobility >200 000 cm^2^/(V s)^41^ and large breaking strength >10%^38^ perfectly fulfils these requirements. By using large area CVD graphene, we can fabricate many devices on a single chip. Finally, the device needs to host a large enough number of unit cells with sufficient periodicity to form a well-defined PnC. While this task is simple in thick SiN, it is much more challenging for fragile freestanding monolayer graphene. To overcome this, we choose a much smaller unit cell compared to typical SiN-PnCs (∼100 μm size) and use helium FIB-milling to pattern the PnC.^42^ This direct lithography allows one to pattern graphene down to 10 nm features,^43^ while causing little damage.^44,45^ A patterned prototype monolayer graphene PnC is shown in Figure 1A. It consists of a honeycomb lattice of holes (lattice constant *a* = 350 nm, hole diameter *d* = 105 nm) around a central region. Within its 10 μm diameter, the two-dimensional PnC contains more than 30 unit cells. The honeycomb lattice inspired by Tsaturayn et al.^15^ exhibits a robust bandgap^12,15,46^ while retaining a relatively large fraction of material to ensure a stable device. Additional PnC with various patterning sizes are shown in Figures S1–S3.

Next, we map the tension within the produced structures using Raman spectroscopy. We expect tension hot spots in the thin ribbons and relaxation in the centers of the hexagons.^47^ Such tension redistribution should affect the vibrational properties of our PnC. To this end, we fabricate another prototype device (Figure 1B) with lattice constant *a* = 2 μm and spatial features comparable to the size of a focused laser spot. The intensity map of the 2D-Raman mode of graphene for this device is shown in Figure 1C. The intensity of the 2D-mode corresponds to the amount of material while its spectral position depends on the tension in the material.^48,49^ In the pizza-like image, one can clearly see the removed material from the drop in intensity and identify the honeycomb lattice. In Figure 1D, we compare the spectral position of the Raman 2D-mode for a graphene PnC (blue) along the dashed line shown in Figure 1C to an unpatterend graphene membrane (red). The quasi-periodic variations in the PnC device that are absent in the unpatterned reference correspond to the redistributed tension. In Figure 1E, we compare the extracted relative tension (blue) to a simulation (yellow) and find the expected signatures of tension redistribution, that is, higher tension between the holes and lower tension in the middle of the hexagons (details in Supporting Information).

### Phononic Crystal Simulations

Having experimentally established the feasibility of a suspended graphene PnC, we use our findings to simulate its phononic properties in two independent approaches. First, we calculate the phononic band structure for an infinitely repeated unit cell (“infinite model”). This model is well-accepted and fast.^15−17^ However, due to the size limits of suspended graphene, our devices are smaller than typical SiN-PnCs (mm size)^15−17^ and contain fewer unit cells. Furthermore, we want to apply pressure to the entire system and investigate localized modes in the bandgap. Therefore, we also simulate a more realistic system of finite size (“finite model”). For both models, we use the honeycomb lattice with feasible parameters and account for tension redistribution upon fabrication (Figure 1D,E). We choose a lattice constant *a* = 1 μm, a filling factor of *d*/*a* = 0.5 (slightly larger than in Figure 1), and an initial tension of *T*_0_ = 0.01 N/m, which is a realistic value for clean monolayer graphene.^39,50^

### Infinite Model

**Figure 2:**
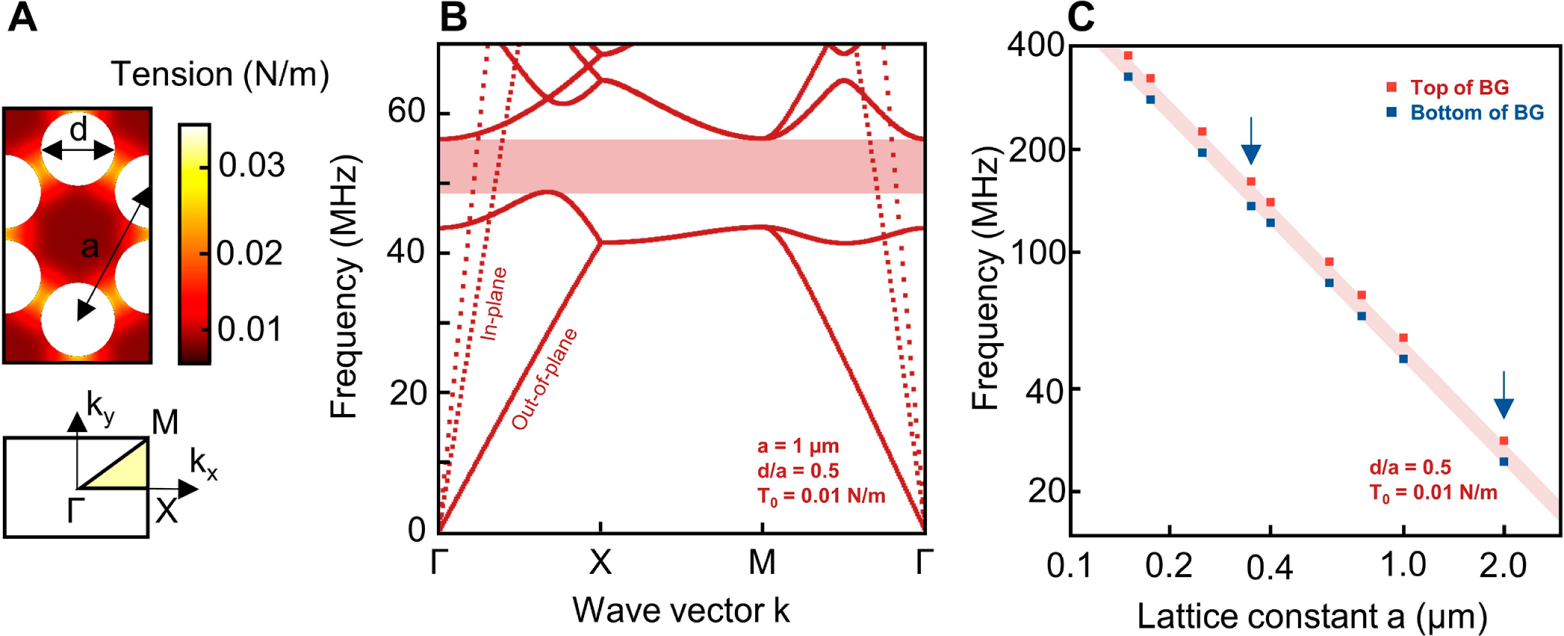
Band structure calculations of an infinite graphene phononic crystal. (A) Unit cell of the honeycomb lattice with redistributed tension (top) and the corresponding first Brillouin zone (bottom). (B) Phononic band structure for the unit cell shown in (A). In-plane modes are shown as dashed lines, out-of-plane modes as solid lines, and the corresponding quasi-bandgap region as the red-shaded area. (C) Top (red) and bottom (blue) of the bandgap versus lattice constant. The blue arrows indicate the lattice constant of the devices from Figure 1.

By applying periodic boundary conditions to the unit cell (Figure 2A), we calculate the band structure for an infinite honeycomb lattice (Figure 2B). We find a mixture of in-plane (dashed lines) and out-of-plane modes (solid lines). From the slope of the out-of-plane modes in Figure 2B, we determine the speed of sound  83 m/s. In the range from 48.8 to 56.5 MHz (red shaded area), we find a bandgap for out-of-plane modes. This quasi-bandgap (in-plane modes are still present) has a gap-to-midgap ratio of 14.6%. The in-plane modes do not couple to out-of-plane modes^51^ and therefore do not hinder radiation shielding. The bandgap originates from Bragg scattering, with each hole acting as a scatterer for out-of-plane oscillations. Upon negative interference conditions, directional Bragg bandgaps open at the high symmetry points. Where these gaps overlap, radiation shielding becomes possible, as wave propagation is isotropically forbidden.^1^ The bandgap position depends reciprocally on *a*. With our fabrication schema, we can tailor the bandgap center from 350 to 26 MHz by varying *a* from 0.175 to 2 μm (Figure 2C, devices in Figures S2 and S3). Overall, the simulations in the infinite model suggest the possibility of a large quasi-bandgap, which we will next use to control phonons.

### Finite Model

**Figure 3:**
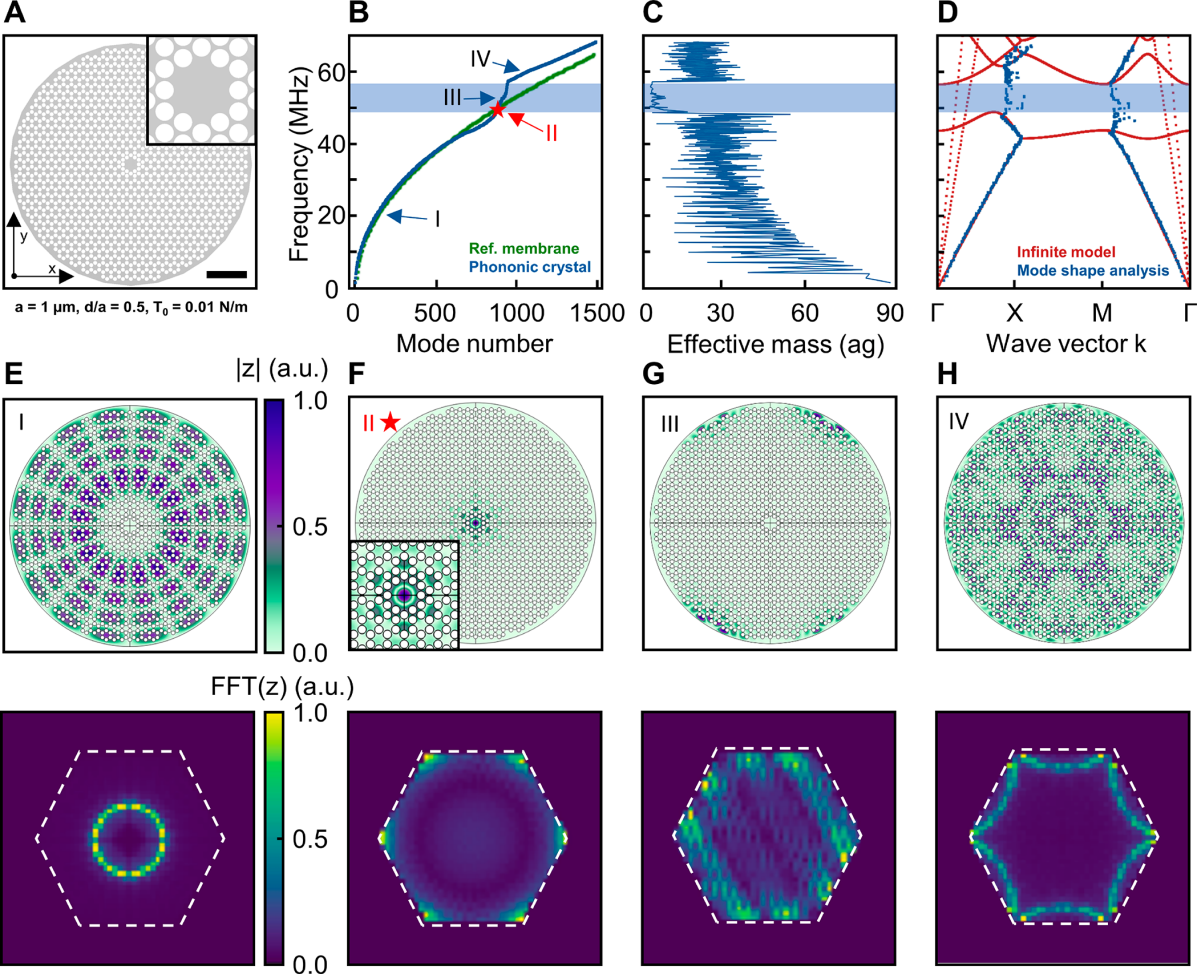
Finite size model of a graphene phononic crystal. (A) Device geometry for the finite system simulations (scale bar is 5 μm). A central “defect” region is designed to localize one vibrational mode and decouple it from its environment. (B) The first 1500 simulated eigenfrequencies versus mode number for a PnC device (blue) and a circular membrane without patterning (green). The bandgap region from the infinite model is shown in blue. (C) Effective mass for each mode. The modes within the bandgap (blue) show a more than a 100-fold decrease in effective mass compared to the fundamental mode. (D) Band structure calculated from the finite model via mode-shape analysis (blue) along with the band structure from the infinite model (red). The low-energy acoustic branches fit well, and the bandgap regions coincide with the simulated results from the infinite model (red). (E–H) Exemplary mode shapes in real (top) and reciprocal space (bottom) for (E) a mode below the bandgap (I), (F) the defect mode (II), (G) another highly localized mode in the bandgap (III), and (H) a mode above the gap (IV).

To study a realistic device of finite size under electrostatic pressure and to implement a defect into the phononic pattern, we conduct a second independent simulation (“finite model”). In this model, we consider a finite number of unit cells of the honeycomb lattice (same *a*, *d*/*a*, and *T*_0_ as before) and employ fixed boundary conditions along the PnC’s perimeter. We choose a circular device as such a geometry allows uniform suspension and minimizes edge effects. In the center of the 30.6 μm device, we create a 1.9 μm hexagonal defect,^15^ as sketched in Figure 3A. Freestanding graphene devices of that size have been fabricated^52^ and the central defect area is large enough to measure resonances interferometrically.^53,54^ Next, we simulate the first 1500 eigenfrequencies and the corresponding spatial mode shape. In Figure 3B, we plot the frequencies *f* versus mode number *N* for the PnC (blue) and compare it to an unpatterned graphene membrane as reference (green). The graph for the PnC shows signs of a bandgap, as we observe an initial flattening of the curve followed by a sudden increase. This region of reduced mode density coincides exactly with the bandgap from our infinite model (blue area) and stands in contrast to the unpatterned membrane for which the frequencies gradually increase with mode number. The second indication of the bandgap is evident when we examine the effective mass of the modes
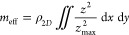
where ρ_2D_ is the areal density of graphene and *z* (*z*_max_) is the (maximum) vibration amplitude in *z*-direction. For the fundamental mode we obtain *m*_eff_ = 80.9 ag = 0.252 m_physical_, which roughly matches the literature value for the mode shape of a uniform, circular membrane of m_eff_ = 0.269 m_physical_.^55^ We observe a pronounced drop of *m*_eff_ in the bandgap region (Figure 3C). This observation is consistent with localized modes inside the bandgap, which typically show a small average displacement resulting in a reduced effective mass.^17^

Finally, we directly extract the band structure from the results of the finite model and compare it to that of the infinite model. To accomplish this, we analyze the mode shape of each resonance following ref (56). Specifically, we take the spatial FFT of each mode shape to find its representation in reciprocal space and to assign a wave vector *k* to each mode. In Figure 3E–H, we show real space (top) and reciprocal space (bottom) plots of exemplary modes. Mode I (20.2 MHz, Figure 3E) is below the bandgap and resembles a higher order Bessel mode in real space, which transforms to a near-uniform circle in momentum space. A higher frequency mode IV (60.7 MHz, Figure 3H) is situated above the bandgap. For this mode, we observe zone folding as the mode reaches out beyond the 1.BZ (dashed line). Analyzing all 1500 modes lets us restore the dispersion relation beyond the 1.BZ (Figure 3D, blue), which almost perfectly matches the band structure from the infinite model (red). From our observations of reduced mode density (Figure 3B), drop in effective mass (Figure 3C), and mode shape-analysis (Figure 3D), we confirm the presence of a bandgap for out-of-plane modes in a realistic system of finite size.

Next, we examine the modes located within the bandgap and identify the defect mode. In Figure 3G, we show a typical bandgap mode in real (top) and *k*-space (bottom). As most modes in the bandgap, this mode is localized at the edges of the PnC in the real space. However, one mode at frequency 49.9 MHz is localized at the central defect (Figure 3F) and surrounded by the phononic pattern. We therefore identify it as our defect mode of special interest. The *m*_eff_ of the mode is 0.724 ag, which is more than a factor of 100 smaller than the fundamental mode of the system and orders of magnitude lower than for any reported SiN defect mode.^15−17^ Overall, our model confirms the vibrational bandgap for a system of finite size and a localized defect mode within that bandgap.

### Phononic Crystal Tuning

**Figure 4:**
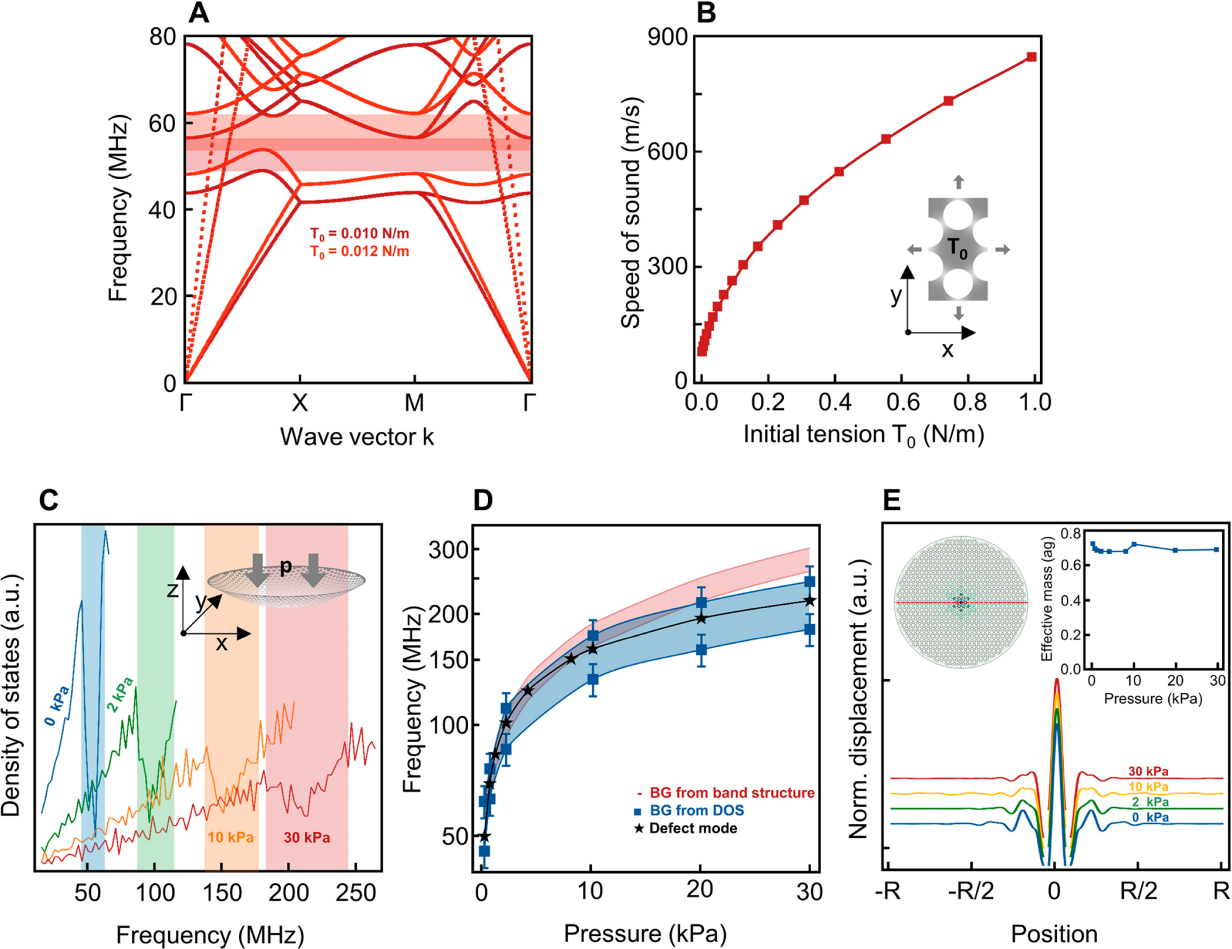
Mechanically tunable graphene phononic crystal. (A) Band structure for initial tension values *T*_0_ = 0.010 N/m (red) and *T*_0_ = 0.012 N/m (orange). The entire out-of-plane branch scales strongly with tension. The position and width of the bandgap are equally tension-dependent. (B) Speed of sound for the out-of-plane modes extracted from (A) versus tension. (C) Density of states calculated from the finite model as a function of pressure applied to the suspended PnC (*T*_0_ = 0.010 N/m). (D) Pressure dependence of resonance frequency of the central defect mode (stars), of the bandgap from infinite model (red shaded), and of the bandgap extracted from the density of states (blue squares). The defect mode remains within the bandgap even at high pressures. (E) Line cut for the spatial profile of the defect mode at different pressures (vertically offset for clarity). Even at large applied loads, the mode shape remains localized, and the effective mass (inset) stays constant.

We now show the key advantage of our graphene PnC: dynamic and rapid frequency tuning of the bandgap and of the defect mode. To demonstrate this, we model our graphene PnC under pressure, which is applied by an electrostatic gate. The pressure causes displacement of the suspended membrane and increases the in-plane tension. We initially approximate this effect in first order in our infinite model by neglecting out-of-plane displacement and simply increasing the in-plane tension. In Figure 4A, we plot the band structure for *T*_0_ = 0.010 and 0.012 N/m. We observe a frequency increase of the out-of-plane modes and thus an upshift of the quasi-bandgap by 10%. The speed of sound *v*_g_ rises from 83 to 830 m/s in the range of tension from 0.01 to 1 N/m (Figure 4B). The system (finite and infinite) behaves like a thin membrane under tension, and the frequencies of the PnC scale directly with tension: .^55^ This scaling makes our system highly sensitive to tension and in combination with graphene’s mechanical flexibility allows for broad frequency tuning.

Having demonstrated the overall tunability of our system, we now simulate the effect of electrostatic pressure on the phononic system and the defect mode in a realistic device. To do so, we switch to the finite model and apply pressure in negative *z*-direction. In our simulations, we stick to experimentally reported pressure values and apply a maximum of 30 kPa.^39^ To investigate the influence of pressure on the bandgap, we compute the phononic density of states, DOS = d*N*/d*f*, and plot it versus pressure (Figure 4C). In this plot, the bandgap is distinguished by a reduced DOS. While at zero pressure the bandgap region is obvious, for higher pressures the drop becomes less pronounced (Figure 4C). We attribute this smearing to a breaking of symmetry, perturbation of the PnC as it deforms under pressure (inset Figure 4C), and rising nonuniformity in the tension distribution (Figure S6E). Nevertheless, we estimate the top and bottom of the bandgap, Figure 4D (blue). A bandgap tuning by more than 300% is evident. We verify the bandgap tuning by an independent approach based on averaging the induced tension (red markers, details in Supporting Information).

Next, we investigate tunability of the defect mode. Upon applying 30 kPa pressure to a device with an initial tension of 0.01 N/m, the resonance frequency of the defect mode upshifts from 49.9 to 217.5 MHz (black stars Figure 4D). Because the bandgap is smeared under pressure (Figure 4C), it is important to check the localization of the defect mode. Hence, we inspect a line cut through the center of the device and plot the normalized mode shape versus pressure in Figure 4E. The shape as well as the effective mass (inset Figure 4E) of the mode remains virtually unchanged and the mode retains its localization. Summarizing, we have shown a tunable speed of sound and realized an upshift of the defect mode resonance under pressure, while maintaining its localization. Such a more than 4-fold frequency increase is unprecedented and remains elusive in any other phononic systems.^22−33^

## Discussion

We now discuss experimental signatures of this system. The spatial features of the extended modes in our device (Figure 3E,H) are too fine to be resolved via diffraction-limited optics. At the same time, the extent of the defect mode is in the size of microns (Figure 3F) allowing the detection of that mode via interferometric read-out (Figure S8).^53,54^ This mode has a nonzero net displacement and can be directly actuated via electrostatic drive. It will be straightforward to distinguish the defect mode from other modes by its localization in the center of the device and its likely increased quality factor. Indeed, the quality factor is defined by *Q* = 2*πE*_stored_/*E*_diss_, where *E*_diss_ is the dissipated energy per oscillation including all dissipation mechanisms and *E*_stored_ is the mode’s total energy. As the mode shape shows zero displacement near the clamping points, we expect strongly suppressed bending losses and thus enhanced *Q*’s. Additionally, the phononic shield hinders radiation losses into the substrate, which become especially important at higher frequencies.^16^ While bending and radiation losses may play a secondary role among the mechanisms lowering *Q* in graphene resonators, our experiments nevertheless should determine the contribution of these mechanisms. Finally, by applying pressure we increase the stiffness of the resonator. This increases the energy stored in the system^17^ and supposedly further enhances the quality factor. The demonstrated level of strain control in our system invites future studies on dissipation dilution via strain engineering following the work of Ghadimi et al.^17^

We also note that our results can be easily extended to the entire family of two-dimensional materials. Currently, it is challenging for us to experimentally achieve sufficient uniformity in the graphene membrane in order to generate a spatially uniform bandgap and localize the defect mode. Monolayer graphene is rather sensitive to surface corrugations^39^ and transferred CVD graphene is often covered by fabrication residues, so using thin exfoliated graphene multilayers could be a solution for which we expect to find experimental signatures. The increased uniformity in multilayer graphene comes along with a decreased tunability, yet we anticipate more than 100% relative tuning for up to ∼35 layers (Figure S9). For our graphene PnC, we do not expect to reach *Q*’s comparable to SiN. Nevertheless, we estimate *m*_eff_ of our defect mode to be at least 8 orders of magnitude lower than in other 2D-SiN-PnCs.^15^ This immensely increases the measurement rate of quantum states Γ_meas_ ∝ 1/*m*_eff_ and decreases thermomechanical noise.^15^ The frequencies in our system are controlled by simply adjusting a gate voltage, and we expect the tuning to take place on time scales comparable to regular graphene resonators and therefore achieve tuning bandwidths >15 kHz.^57^

## Conclusion

In summary, we have fabricated and simulated a tunable PnC made from monolayer graphene. For an experimentally informed honeycomb lattice structure, we find a robust vibrational bandgap in the megahertz range. The bandgap persists for a finite-size system, and we use it to localize a defect mode and shield it from its surroundings. This defect mode shows a very small effective mass of 0.724 ag, orders of magnitude smaller compared to traditional PnCs. As our central result, we demonstrate a frequency upshift of the defect mode as well as the entire phononic system by more than 350% by applying an experimentally feasible pressure of 30 kPa. While the bandgap smears out due to out-of-plane displacement perturbing the lattice, the defect mode stays within the bandgap and remains highly localized. We suggest experimental signatures of the defect mode allowing its differentiation from other modes in the system. Overall, our design of a 2D-material-based PnC adds a new knob to dynamically and rapidly tune frequencies in a broad range of phononic applications. Our results invite future experiments as our approach allows adjustable coupling of a PnC to external systems and may lead to better understanding of the dissipation mechanisms in graphene.

## Methods

### Device Fabrication

The pattering of the CVD grown graphene membranes was carried out in a He-ion microscope (Orion Nanofab). Supporting Information Section I provides a detailed process description.

### Raman Spectroscopy

Raman mapping was performed on a Horiba Xplora Raman spectrometer using a 100× (NA 0.9) objective and 532 nm excitation. Spectra were acquired with a laser power of 0.5 mW and an integration time of 3 s. Tension (via strain) values were derived from the 2D-mode position following standard procedures, see Supporting Information Section IV.

### Simulations

For the finite element modeling we use COMSOL Multiphysics (Version 5.5) and assume the following material parameters for monolayer graphene: Young’s modulus *E*_2D_ = 1.0 TPa,^38^ Poisson’s ratio of *v* = 0.15, thickness of *h* = 0.335 nm, and a density of  2260 kg/m^3^. The initial tension *T*_0_ = 0.01 N/m thus corresponds to an initial strain: . For details see Supporting Information, Sections II and III.

## Supporting Information Available

Graphene pattering using He-FIB milling, details of the FEM-simulations and the mode shape analysis, detailed Raman spectroscopy analysis, and simulations on experimental signatures (PDF)
