# Supplementary material for: Tunable Graphene Phononic Crystal
Source: Nano Lett. 2021 Feb 23;21(5):2174–82. doi: 10.1021/acs.nanolett.0c04986 (PMC7953378; doi:10.1021/acs.nanolett.0c04986)
Supplement: Supplementary file 1 — nl0c04986_si_001.pdf [file nl0c04986_si_001.pdf]

Supporting Information: Tunable graphene phononic crystal

Jan N. Kirchhof[1], Kristina Weinel[1,2], Sebastian Heeg[1], Victor Deinhart[2,3], Sviatoslav Kovalchuk[1], Katja Höflich[2,3] and Kirill I. Bolotin[1*]*

[1]Department of Physics, Freie Universität Berlin, Arnimallee 14, 14195 Berlin, Germany.

[2]Ferdinand-Braun-Institut gGmbH Leibniz-Institut für Höchstfrequenztechnik, Gustav-Kirchhoff-Str. 4, 12489 Berlin, Germany

[3]Helmholtz-Zentrum Berlin für Materialien und Energie, Hahn-Meitner-Platz 1, 14109 Berlin, Germany.

*jan.kirchhof@fu-berlin.de *kirill.bolotin@fu-berlin.de

I. Sample synthesis and device pattering using He-FIB milling

Monolayer graphene was synthesized on copper by low pressure chemical vapor deposition (CVD). Upon reaching the growth temperature of 1035 °C a mixture of methane (5 sccm), hydrogen (10 sccm), and argon (5 sccm) was introduced into the CVD chamber. After 7 min growth time and a rapid cooldown, graphene was wet transferred onto a perforated SiN membrane, covered by Cr/Au (5 nm/35 nm) to electrically contact the graphene.

To pattern the suspended graphene, we used a beam of focused helium ions in the Orion Nanofab microscope. The local material removal upon ion beam impact is a complex interplay between physical sputtering (ions kick out surface atoms), the redeposition of the sputtered surface atoms in the close vicinity, chemical reactions (like polymerization of organic residues by the generated secondary electrons) as well as the introduction of heat and amorphization.[1] In case of two-dimensional material only physical sputtering contributes. The holes were patterned with a dwell time of 1.5 ms and a pixel spacing of 1 nm at a beam current of 4-5 pA (device settings: $2x10^{-6}$ Torr He , U_{acc} = 30 kV, UBIV = 34 kV, aperture 2 µm). The holes on the outside of device were cut first, following a spiralling milling strategy to the centre of the suspended area (Figure S1B). Here each single hole is milled in an opposite spiral order – starting at the centre of the hole (Figure S1C). If the graphene layer is completely intact and free of contamination, the process is highly reproducible (see Figure S2). In Figure S3A we show fabricated phononic crystal devices with varying lattice constant a: 0.175…2 µm. While the patterning allows for highly flexible variation of geometrical parameters, like lattice constant and hole diameter, the process of He-ion induced physical sputtering is highly sensitive to surface contamination. In Figure S3B this effect becomes visible by the bright regions around the

spot. Here, the secondary electrons induced polymerization of the organic residues and therefore material build-up (deposition). The amount of contamination increases the minimum dose to achieve a full cut and may even dominate over physical sputtering as shown here for the smallest spots. As the coverage of organic residues may locally vary also the required minimum dose for complete graphene removal can vary locally. This can be seen in Figure 1B of the main manuscript. Holes with sizes down to 5 nm can be fabricated by He ion beam milling in relatively uniform and uncontaminated monolayer graphene (Figure S3B).

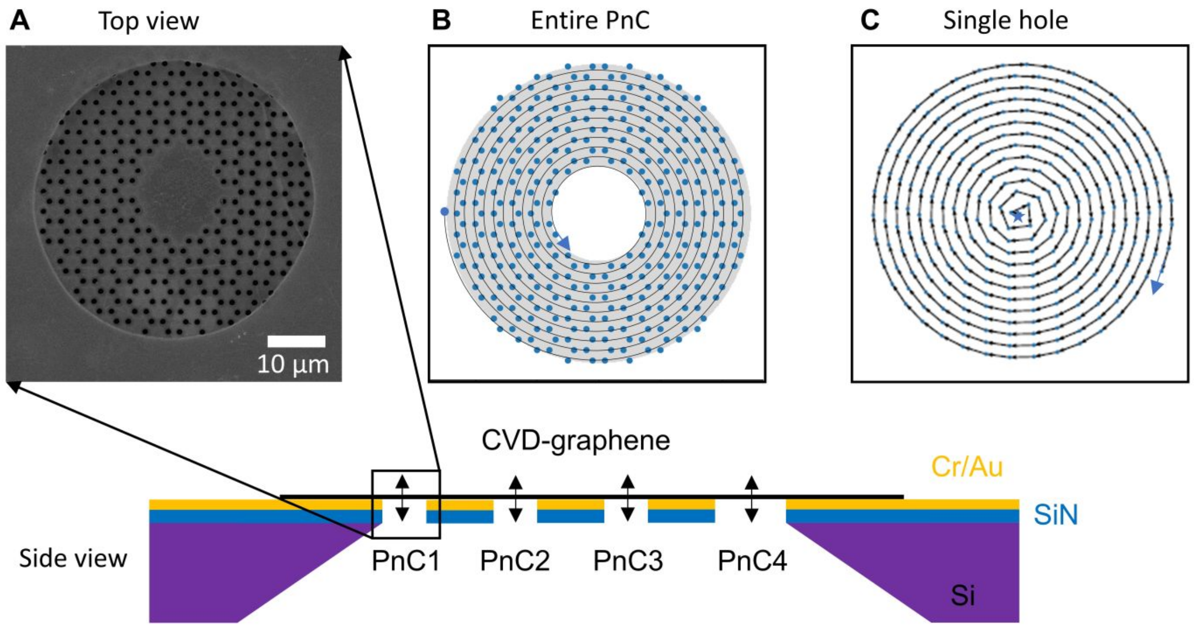

Figure S1. Milling strategy for PnC-device patterning. (**A**) Device with lattice constant *a* = 700 nm. By transferring CVD graphene onto pre-pattered substrates with many through holes, many PnC can be milled on a single chip. (**B**) Corresponding design file including the pattering order – starting at the outside and spiralling towards centre of the device. (**C**) Patterning of a single hole – starting in the centre moving to the outside.

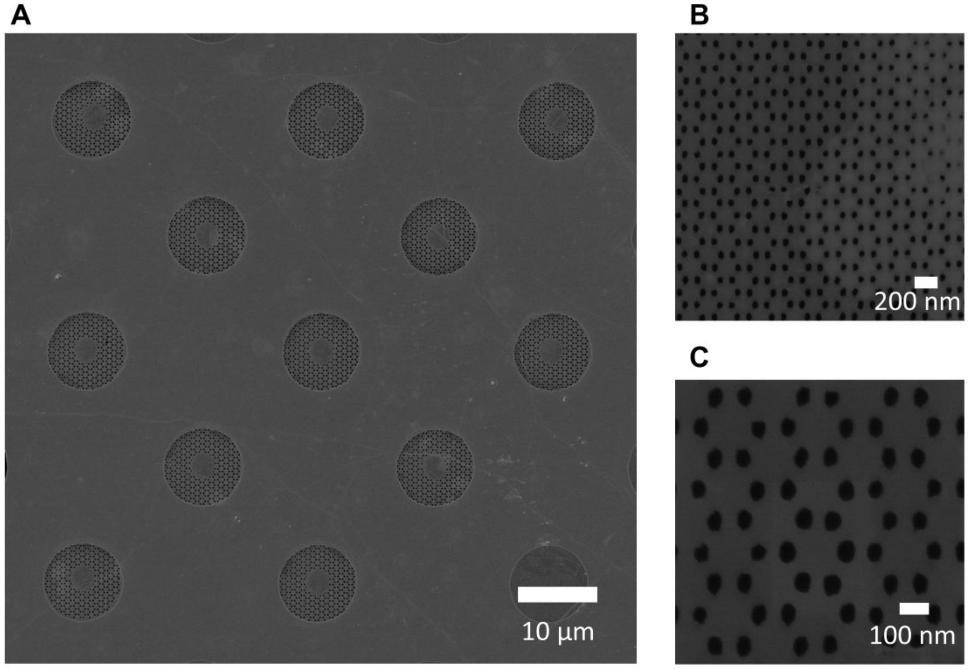

Figure S2. Reproducible PnC patterning. (**A**) Repeated pattering of a device with lattice constant a = 700nm. For uniform graphene the process is highly reproducible. (**B**,**C**) Zoom-in on the honeycomb lattice with a = 175 nm. The milling process is less efficient on add-layer regions – visible on the right half in (**B**).

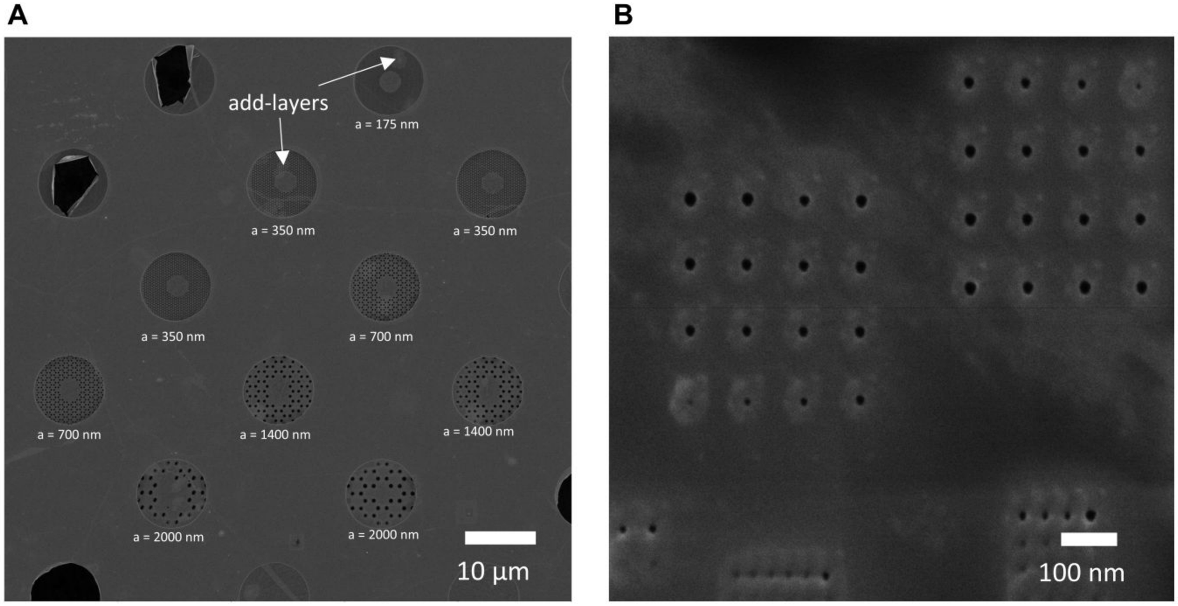

Figure S3. Scalable PnC patterning. (**A**) PnC devices of varying lattice constant a = 175 nm - 2 µm. Occasional add-layer regions from CVD growth are marked. (**B**) Dose tests on uniform and clean graphene, show that pores with sizes well below 5 nm can be fabricated by He ion beam milling.

II. Finite element method simulations

We use the solid mechanics module of Comsol Multiphysics (Version 5.5) to carry out the FEM-simulations presented in the main paper.

The **infinite model** for the band structure calculations consist of two studies within one model. We use a large square containing many unit cells of the phononic pattern and implement uniform tension in the solid (Figure S4). In a stationary study with fixed boundary conditions at the edges, we simulate the tension redistribution, which occurs upon cutting holes into a system under tension.

We then add a second study (eigenfrequency domain) within in the same model to simulate the resonances und thus plot the band structure. To accurately depict the tension distribution, we crop the central unit cell of the large square from the first study and component-wise transfer the tension distribution to the second study (Fig S4). To obtain the band structure, we apply periodic boundary conditions (Floquet) to the edges of the unit cell and parameterize k_x and k_y (in an auxiliary sweep) along the high symmetry lines in the first Brillion zone and calculate the first 6-10 eigenfrequencies for every value of k. By plotting the frequencies f vs. k, we get the dispersion relation for the geometry of interest. We use a swept mesh as we simulate a very thin system. Furthermore, we apply copy operators within in the unit cell when building the mesh to completely capture the symmetry of the system (Fig S5B). In general, the size of the bandgap depends on the filling factor d/a. Choosing d/a~0.5 (slightly larger than for Figure 1 in the main paper) results in a reasonably sized bandgap, whilst leaving behind enough material to reproducibly fabricate devices. In Figure S5, we provide a detailed study of bandgap width vs. d/a. Taking into account for tension redistribution overall reduces the size of the bandgap. For the second estimate of the bandgap tuning with applied pressure (main text,

Figure 4D) we extract the average tension in the finite model at each pressure value and feed the average values as input into our infinite model.

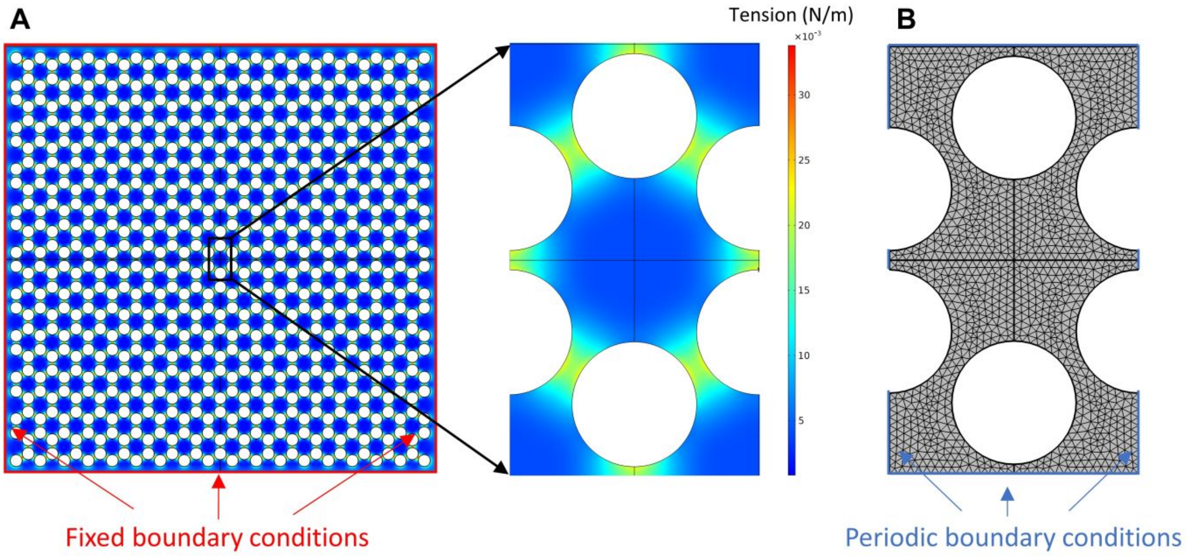

Figure S4. Infinite model PnC simulations. (**A**) A large membrane is needed to correctly calculate the tension redistribution. A central unit cell is cropped and used for the band structure calculations (**B**) Corresponding mesh of the unit cell.

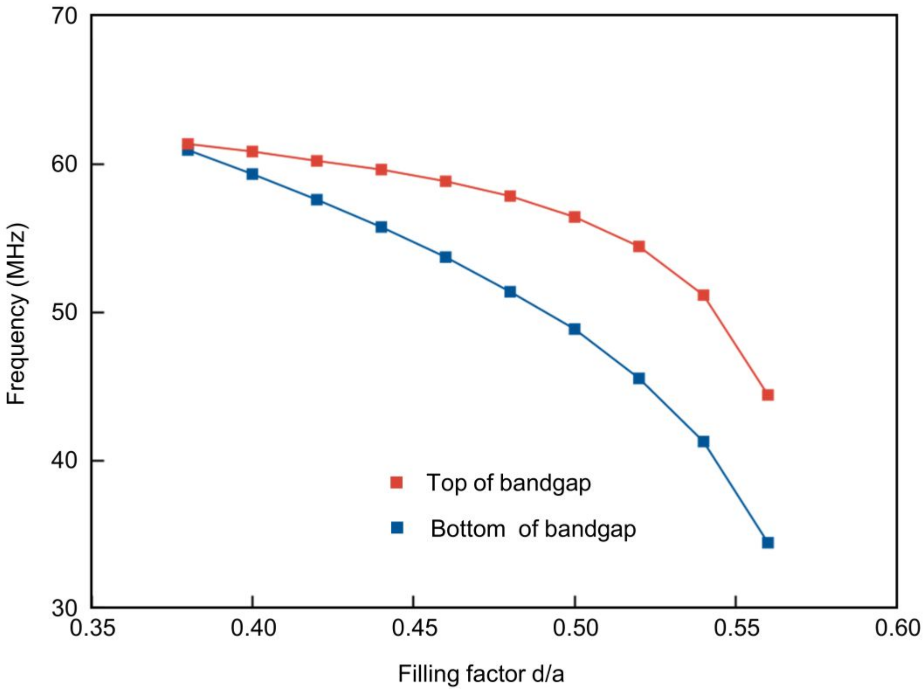

Figure S5. Phononic Bandgap vs. filling factor. Frequencies of the top and of the bottom of the bandgap vs. the filling factor d/a for a = 1 μm and an initial tension of T_0 = 0.01 N/m. Tension redistribution is accounted for.

For the calculations in the **finite model** we use a membrane model from the solid mechanics module with two study steps (Figure S6). In the first step, we again let the system relax after adding uniform built in tension. The resulting tension distribution is shown in Figure S6 C and D. We then calculate the first 1500-2500 resonances of the system in an eigenfrequency study step. The mode shapes and frequencies are exported for further analysis in a python script (see section III). Also here it is important for the mesh to represent the symmetry of the modelled geometry – compare Figure 6B.

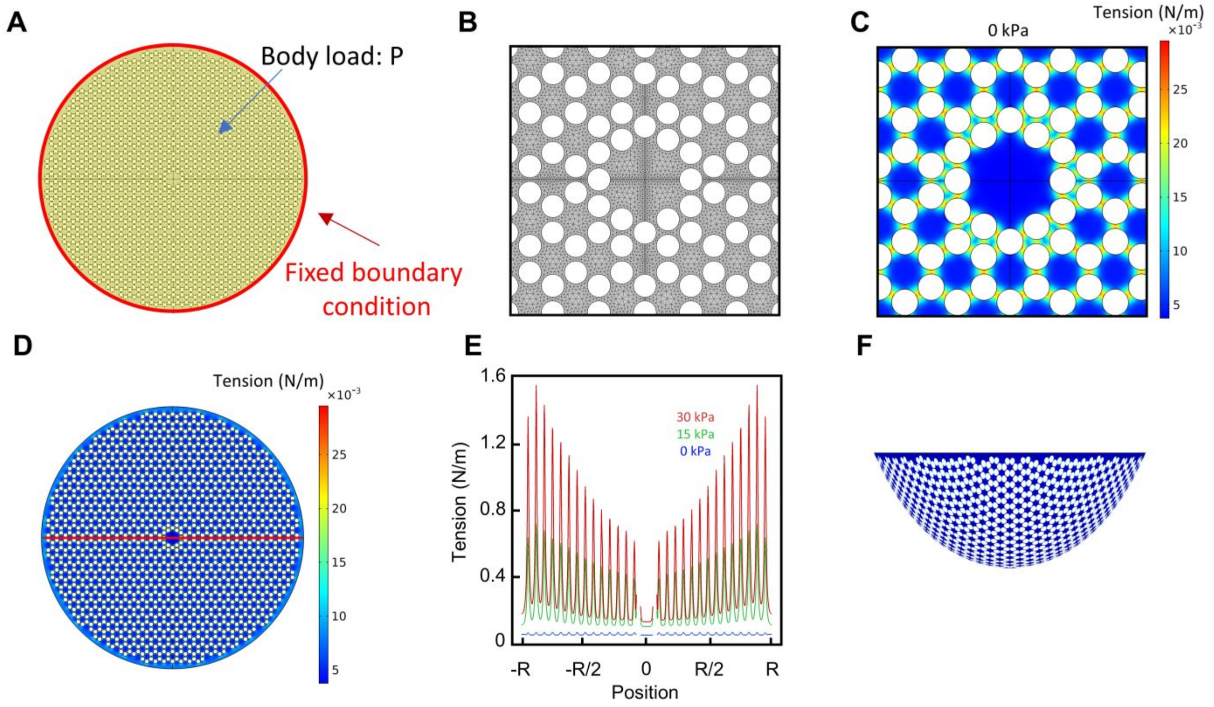

Figure S6. Finite model PnC simulations. (**A**) Circular PnC of 30.6 µm size. The electrostatic pressure is applied as a body load (yellow). (**B**) Symmetric mesh. (**C**) Tension after the redistribution step around the central defect region and in the entire device (**D**). (**E**) Tension along a line cut (marked red in (**D**)) for different pressures. With larger pressures, the distribution becomes increasingly non-uniform. (**F**) Deformation of the finite system under large pressures (100 kPa) to illustrate the perturbation of the phononic pattern.

III. Mode shape analysis

We first export the mode shape for each mode obtained from our Comsol simulations and interpolate it onto a square grid with 1000 nodes. Next, we carry out a fast Fourier transform (FFT) on such an array to obtain the reciprocal space representation of each

mode. Most modes, except for the ones within the bandgap, have a well-defined momentum along each specific direction. To determine the momentum content of each mode, we take a cut of each mode in the momentum space and find a peak along each particular direction. To aid in this procedure and reduce noise, we average over 10 neighboring modes. Knowing the momentum, we finally export the dispersion relation along the direction of interest.

IV. Raman spectroscopy analysis

In the main paper, we use initial tension T_0 as a device parameter to avoid confusion. For Raman data however it is more common characterize graphene in terms strain ϵ_0, which is directly linked to tension value via the 2D-Youngs-modulus $T_0 = \epsilon_0 E_{2D}$. In this section, we discuss in detail the signatures of strain redistribution obtained by Raman spectroscopy of the graphene phononic crystal presented in the main paper. Figure S7A shows a Raman map of the integrated 2D-mode intensity of the suspended membrane, see Figure 1D of the main paper. The holes forming the phononic crystal are clearly marked by a local decrease in 2D-mode intensity. We show a representative Raman spectrum from the centre of the phononic crystal in Figure 7B, marked by (#) in Figure S7A. The experimentally observed intensity ratio between the Raman G and D peaks, I(2D)/I(G)>1, proves that the phononic crystal is made from a single layer of graphene. We used SEM imaging to avoid measuring the devices with bilayer graphene areas (Fig. S3A). The appearance of the D, D' and D+D' mode indicate the presence of defects, which arise mainly from repeated electron beam imaging of the graphene membrane.

To demonstrate the onset of strain relaxation, we focus on a horizontal line cut (along x within out laboratory frame) across the phononic crystal at y = 2.3 μm as indicated by the dashed line in Figure S7A. Figure S7C shows the corresponding integrated 2D-mode

intensity (squares) and position (triangles) as a function of x, where the origin (0,0) was set at the centre of the membrane. We observe four equidistant drops in intensity, indicated by arrows, which corresponds to the narrow graphene stripes between the holes (compare to Figure S7A). The drop in intensity occurs because at the strips, the laser spot overlaps with the holes in the graphene membranes such that less material is probed compared to regions further away from the holes. For the two narrow graphene stripes closest to 0, we find that the 2D-mode position drops by 3-5 cm^{-1} compared to the central region of the graphene membrane. As hydrostatic strain lowers the energy of phonons probed by Raman spectroscopy,[2,3] our observation suggests that the narrow stripes carry a higher strain then neighbouring parts of the graphene phononic crystal, which is in qualitative agreement with the strain redistribution introduced in the main paper. A similar but less pronounced behaviour occurs for the two additional narrow stripes closer to the edge of the suspended graphene membrane at $x\sim \pm 3\mu m$, where the local decrease in 2D-mode position (increase in strain) is overlaid with a general increase of the 2D-mode position (decrease in strain) towards the edge of the suspended part of the graphene membrane at $x\sim \pm 4\mu m$. The overall lower position 2D-mode in the centre of the membrane could be due to laser heating. The pattering reduces the thermal conductance of the system and thus even at small powers (0.5 mW) heating can occur.

Next, we quantify the hydrostatic strain in our phononic crystal, which is presented in Figure 1E of the main paper. Hydrostatic strain ε_h in graphene leads to a shift $\Delta\omega_{2D}$ of the 2D-mode position ω_{2D} following the relation.[2,3]

$$\Delta\omega_{2D} = -\,\varepsilon_h\,\gamma_{2D}\,\omega_{2D}^0 \quad (1)$$

where $\gamma_{2D} = 2.6$ is the Grüneisen parameter of the 2D-mode in graphene, and ω_{2D}^0 is the intrinsic 2D-mode position without strain or doping ($\omega_{2D}^0 = 2678\ cm^{-1}$ for $532\ nm$

excitation) [4]. From the measured 2D-position ω_{2D}^{epx} , $\Delta\omega_{2D} = \omega_{2D}^{0} - \omega_{2D}^{epx}$, and equation (1) we extract the strain values from Figure S7C and show them together with the 2D-mode position in Figure 1 of the main paper.

The general trend of lower strain towards the edge of the suspended graphene phononic crystal suggests that strain relaxation is not complete across the entire structure. We attribute this behaviour to two main reasons. First, strain in suspended graphene membranes is never homogeneous, see reference membrane in Figure 1D of the main paper, and the degree of strain relaxation scales with the absolute strain values. Therefore, we do not expect homogenous strain relaxation across the entire phononic crystal. Second, strain in suspended graphene visibly varies on length scales comparable to the size of the holes in the suspended graphene membrane, see Figure 1D of the main paper, which makes strain relaxation less effective. Here we chose a PnC with rather large lattice constant a such that strain variation and relaxation can be probed by Raman spectroscopy with diffraction limited spatial resolution. For phononic crystals with holes sizes and periodicities that are much smaller than the variations of initial strain in suspended graphene structures, we expect strain relaxation to be more efficient than what is observed for the phononic crystal discussed here.

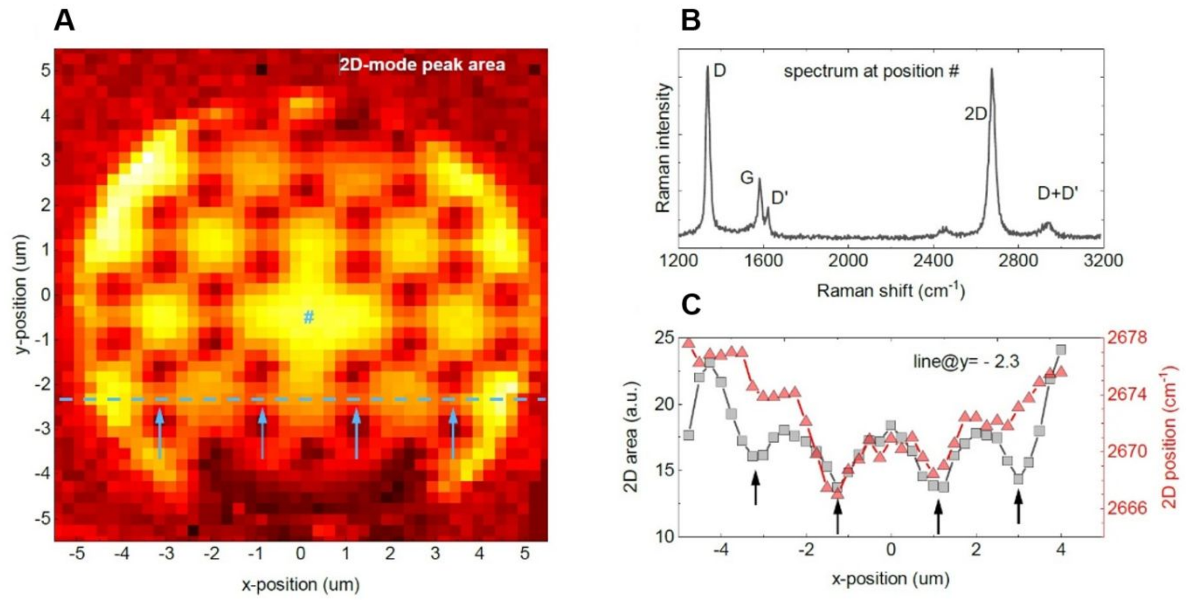

Figure S7. Raman characterization of the graphene PnC. (A) Raman map of the graphene 2D-Raman mode (integrated intensities. Intensity drops mark the locations of the holes in the hexagonal arrangement that forms the phononic crystal. (**B**) Representative Raman spectrum of the graphene membrane extracted at the location marked as (#) in (**A**). The dominant Raman modes of graphene are labelled. (**C**) Integrated 2D mode intensity (area, grey squares) and 2D-mode position (red triangles) along a line cut at y = 2.3um in x-direction as indicated by the dashed line in (**A**). Arrows in (**A**) and (**C**) mark the locations of narrow graphene bridges between the holes where strain relaxation is expected.

V. Experimental signatures of the defect mode

We propose detecting the defect mode by interferometric detection. In this approach a laser beam reflected from the device interferes with a reference beam providing an accurate measurement of membrane's position.[5–8] We need to confirm, however, that diffraction-limited laser spot is small enough to measure signatures of a realistic defect mode. To confirm that the defect mode in the center of the PnC presented in the main text is detectable, we simulate the spatial signal read out by the interferometer by implementing a Gaussian averaged laser spot for a realistic source reflected from our structure. In Figure S8 we show this for multiple laser spot sizes. For the smallest possible spot with a FWHM of 720 nm, even small spatial features of the mode shape are detectable (Figure S8A). For a realistic spot size (FWHM of 2400 nm) including the window of a vacuum chamber and a larger working distance objective we are still able to measure the mode (Figure S8B). And finally, we take a very large spot (FWHM of 7.2 μm) and thereby probe the entire mode shape (Figure S8C). We can confirm that the mode will not be fully averaged out, as it has net displacement (in contrast to e.g. mirror symmetric modes). Overall, we confirm that we should be able to detect the motion of the defect mode for all realistic laser spot sizes.

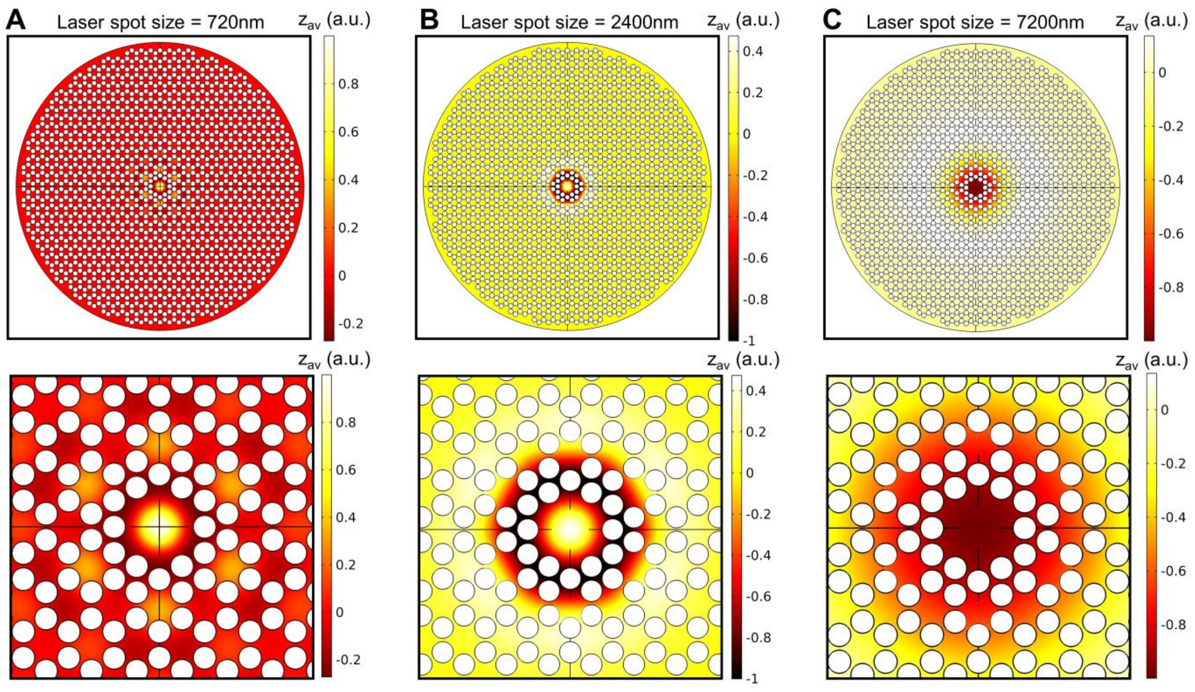

Figure S8. Interferometric detection of the defect mode. (**A-C**) Mode shape of the defect mode with local Gaussian averaging to simulate the displacement detection via a focused laser spot of different width (Zoom-ins are shown below). Different panels correspond to different spot sizes. Even for the largest laser spot size a net displacement is evident.

As mentioned in the main text spatial uniformity is necessary to fabricate a phononic crystal with a well-defined band structure. Monolayer graphene is sensitive so surface effects, wrinkling and fabrication residues. Using multilayer graphene would solve this problem yet will also be less responsive to the experimentally possible pressure maximum of roughly 30 kPa. To check if a PnC made from multilayer graphene would still show frequency tuning, we simulate the resonance frequency of a uniform circular membrane (initial tension 0.01 N/m) with and without applied load of 30 kPa. In Figure S9 we plot the relative frequency change under pressure vs. number of graphene layer. Even though the tunability drops quickly for thicker graphene, we still find more than 100% possible upshift for 35 layers.

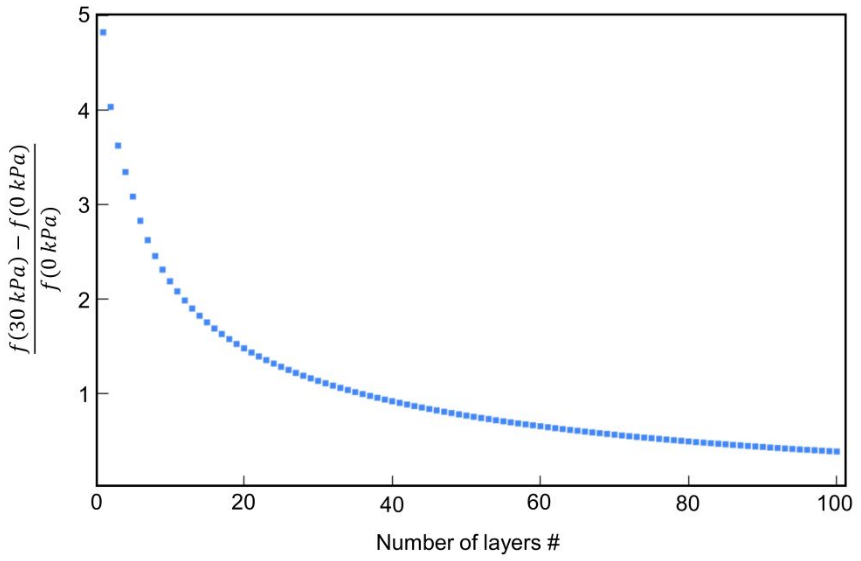

Figure S9. Frequency tunability vs. number of layers. Relative frequency shift of the fundamental mode of a circular multilayer graphene resonator upon applying 30 kPa of pressure vs. number of graphene layers.

References

(1) Utke, I.; Hoffmann, P.; Melngailis, J. Gas-Assisted Focused Electron Beam and Ion Beam Processing and Fabrication. *J. Vac. Sci. Technol. B Microelectron. Nanom. Struct.* **2008**, *26* (4), 1197.

(2) Mohiuddin, T. M. G.; Lombardo, A.; Nair, R. R.; Bonetti, A.; Savini, G.; Jalil, R.; Bonini, N.; Basko, D. M.; Galiotis, C.; Marzari, N.; Novoselov, K. S.; Geim, A. K.; Ferrari, A. C. Uniaxial Strain in Graphene by Raman Spectroscopy: G Peak Splitting, Grüneisen Parameters, and Sample Orientation. *Phys. Rev. B - Condens. Matter Mater. Phys.* **2009**, *79* (20), 1–8.

(3) Mueller, N. S.; Heeg, S.; Alvarez, M. P.; Kusch, P.; Wasserroth, S.; Clark, N.; Schedin, F.; Parthenios, J.; Papagelis, K.; Galiotis, C.; Kalbáč, M.; Vijayaraghavan, A.; Huebner, U.; Gorbachev, R.; Frank, O.; Reich, S. Evaluating Arbitrary Strain Configurations and Doping in Graphene with Raman Spectroscopy. *2D Mater.* **2018**, *5* (1).

(4) Froehlicher, G.; Berciaud, S. Raman Spectroscopy of Electrochemically Gated Graphene Transistors: Geometrical Capacitance, Electron-Phonon, Electron-Electron, and Electron-Defect Scattering. *Phys. Rev. B - Condens. Matter Mater. Phys.* **2015**, *91* (20), 205413.

(5) Singh, R.; Nicholl, R. J. T.; Bolotin, K. I.; Ghosh, S. Motion Transduction with Thermo-Mechanically Squeezed Graphene Resonator Modes. *Nano Lett.* **2018**, *18* (11), 6719–6724.

(6) Singh, R.; Sarkar, A.; Guria, C.; Nicholl, R. J. T.; Chakraborty, S.; Bolotin, K. I.; Ghosh, S. Giant Tunable Mechanical Nonlinearity in Graphene-Silicon Nitride Hybrid Resonator. *Nano Lett.* **2020**, *20* (6), 4659–4666.

(7) Zande, A. M. Van Der; Barton, R. A.; Alden, J. S.; Ruiz-vargas, C. S.; Whitney, W. S. Large Scale Arrays of Single Layer Graphene Resonators. - Supplementary Information. *Nano Lett.* **2010**, *10* (12), 4869–4873.

(8) Bunch, J. S.; Van Der Zande, A. M.; Verbridge, S. S.; Frank, I. W.; Tanenbaum, D. M.; Parpia, J. M.; Craighead, H. G.; McEuen, P. L. Electromechanical Resonators from Graphene Sheets. *Science (80-.).* **2007**, *315* (5811), 490–493.
